# Promoting action of vitamin E and black seed oil on reproductive hormones and organ histoarchitecture of Swiss albino mice

**DOI:** 10.1002/vms3.708

**Published:** 2022-01-17

**Authors:** Afrina Mustari, Mohammed Nooruzzaman, Mohammad Alam Miah, Khaled Mahmud Sujan, Emdadul Hauqe Chowdhury

**Affiliations:** ^1^ Department of Physiology, Faculty of Veterinary Science Bangladesh Agricultural University Mymensingh Bangladesh; ^2^ Department of Pathology, Faculty of Veterinary Science Bangladesh Agricultural University Mymensingh Bangladesh

**Keywords:** black seed oil, folliculogenesis, Johnsen score, ovary, testes, vitamin E

## Abstract

**Background:**

Vitamin E and black seed oil are two powerful antioxidants with several health benefits.

**Objective:**

The effect of vitamin E and black seed oil on reproductive performance of Swiss albino mice was studied.

**Methods:**

A total of 80 (40 male and 40 female) mice of 25–28 days old were randomly divided into four groups viz., A, B, C and D consisting of 10 mice in each group. Mice from the group A served as vehicle control and received normal mice ration whereas mice from the group B, C and D received feed supplemented with either black seed oil (0.5 ml/kg), vitamin E (200 mg/kg) or combination of black seed oil (0.5 ml/kg) and vitamin E (200 mg/kg), respectively daily for 16 weeks. At the end point of the study, blood samples were collected and sera were separated for hormonal analysis. At the same time, mice were sacrificed and testes and ovaries were collected for histomorphological examination.

**Results:**

In male mice, the level of testosterone increased significantly in mice receiving black seed oil only, whereas the thyroxin increased significantly in all treated groups when compared to the control mice. Histomorphological examination revealed a significant increase in the diameter of seminiferous tubules in male mice fed with either black seed oil or vitamin E or both. On the other hand, the oestradiol and thyroxin concentration in female mice showed no significant changes in both control and treated groups. However, ovaries of mice fed with black seed oil or vitamin E or both showed an increased number of the follicles of different stages than the control mice.

**Conclusions:**

The findings highlighted the promoting action of vitamin E and black seed oil on reproductive functions of mice and that can be used to treat infertility in man and animals.

## Graphical abstract

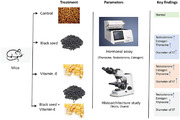

## 1 INTRODUCTION

Exposure to chemicals (such as pesticides or insecticides), heavy metals, high temperature, electromagnetic wave radiation, smoking, stress, alcohol and obesity disrupts the spermatogenesis and folliculogenesis in animals and humans (WHO, 2010). Indicators of spermatogenesis disruption are observed based on sperm quantity, sperm quality and abnormal testosterone levels (Jedrzejowska et al., 2013). The specific cases of infertility are normally corrected by surgery, medication, assisted reproductive technology (ART) and so on. But limitations on infertility treatment are very common and most of the techniques have detrimental effects on health. Therefore, it is a prime concern to find out an alternative way to improve reproductive health.

Free radicals are continuously produced in the body due to metabolic and nutritional deficiencies. A free radical is defined as a reactive oxygen molecule containing one or more unpaired electrons in atomic or molecular orbitals (Tremellen, 2008). Free radicals are chemically unstable molecules that damage cell lipids, proteins and DNA. An imbalance between the generation of reactive oxygen species (ROS) and the activity of antioxidant enzymes enhances damage of these cell components (Lobo et al., 2010). A high level of ROS production leads to peroxidation of the sperm acrosomal membrane and diminishes acrosin activity, and impairs sperm–oocyte fusion (Jedrzejczak et al., 2005). Free radicals have the ability to directly damage sperm DNA by attacking the purine and pyrimidine bases and the deoxyribose backbone (Geva et al., 1998). If ROS production increases, it results in damage to the DNA, endothelial destruction and testicular germ cell apoptosis (Karaguzel et al., 2014; Moghimian et al., 2017). Moreover, higher ROS could damage the mitochondrial membrane and stimulate the release of cytochrome C, leading to the initiation of the intrinsic apoptosis pathway in testicular tissue cells (Chresta et al., 1996; Shokoohi et al., 2020).

About 30% of all modern drugs are derived from plant sources (Burns, 2000). The seeds of *Nigella sativa* Linn. (Ranunculaceae herbaceous plant), commonly known as black seed or black cumin are being used as herbal medicine all over the world having gastroprotective, anti‐tumour, anti‐anxiety, antimicrobial, anti‐inflammatory and anti‐oxidant properties (Kanter et al., 2003). The seeds of *N. sativa* have many different chemical components including mucilage, crude fibre, reducing sugars, resins, alkaloids, flavonoids, organic acids, sterols, tannins, saponins and proteins (Swamy & Tan, 2000). In addition, it has a high content of unsaturated fatty acids, especially linoleic acid, oleic acid and palmitic acid (Nickavar et al., 2003). Much of biological activity of the seeds has been attributed to thymoquinone (30%–48%), the major component of the essential oil, but it is also present in the fixed oil (Burits & Bucar, 2000). Antioxidant property of thymoquinone is attributed to the quinine structure of thymoquinone molecule (Padhye et al., 2008) and the easy access of the thymoquinone to subcellular compartments facilitates the ROS scavenging effect (Badary et al., 2000). Thymoquinone has been shown to inhibit nonenzymatic lipid peroxidation which leads to decreased oxidative stress and protection of the antioxidant enzymes of the testis (Ismail et al., 2010). Black seed have an active effect on reproductive functions and infertility treatment demonstrated significant improvements in respective factors, including sperms, semen, leydig cells count, follicular development, corpus luteum and gonadotropic hormones like testosterone and progesterone (Darand et al., 2020). Mukhalad et al. (2009) showed that the aqueous extract of the *N. sativa* increases the spermatogenesis in male albino rats.

Natural antioxidants such as vitamin E are widely used as dietary supplements due to their capacity to protect tissues from oxidative stress caused by ROS (Sreeramulu & Raghunath, 2010). Vitamin E are absorbed through the intestine in presence of other lipid‐rich foods. Following absorption, vitamin E requires vascular transport to the liver mainly with the help of α‐tocopherol‐transfer protein. Metabolism of vitamin E begins with one cycle of CYP4F2/CYP3A4‐dependent ω‐hydroxylation followed by five cycles of subsequent β‐oxidation, and forms the water‐soluble end‐product carboxyethylhydroxychroman. All known hepatic metabolites can be conjugated and are excreted, depending on the length of their side chain, either via urine or faeces (Schmölz et al., 2016). As an antioxidant, vitamin E acts as a peroxyl radical scavenger, preventing the propagation of free radicals in tissues, by reacting with them to form a tocopheryl radical that is reduced by a hydrogen donor and return to its reduced state. Due to its solubility, it is incorporated into cell membranes, which protects them from oxidative damage (Gokce et al., 2011). The use of vitamin E increases the reproductive functions and efficiency of male reproductive system. In vitro studies have proved that the use of vitamin E improves the motility and fertilizing ability of sperm in hamster (Plante et al., 1994). Riley et al. showed the effect ROS and the antioxidant enzymes in oocyte maturation, ovulation and luteal function (Riley & Behrman, 1991). Similarly, in vivo studies showed that treatment of vitamin E protects ROS‐induced sperm damages and increases the number and motility of sperms (Zubair, 2017).

Few studies have been conducted to evaluate the effect of black seed oil on reproduction function, however, studies on vitamin E and combination of black seed oil and vitamin E are limited. Therefore, the present study was aimed at investigating the effects of black seed oil and vitamin E on the reproductive function of adult male and female Swiss albino mice.

## 2 MATERIALS AND METHODS

### 2.1 Mice

A total of 80 (40 males and 40 females) Swiss albino mice (*Mus musculus*), aged between 25–28 days with an average body weight of 27.4 ± 1 g were received from the International Center for Diarrheal Disease Research, Bangladesh (icddr’ b). Animals were kept under optimal management conditions including temperature, humidity, ventilation and light.

### 2.2 Treatment

The mice were randomly divided into four groups, viz., A, B, C and D consisting of 10 mice in each group for each sex. Group A served as vehicle control and was provided daily oral doses of normal mice ration. Group B mice were administered with black seed oil daily oral doses (0.5 ml/kg), while group C received vitamin E daily oral doses (200 mg/kg) and group D was administered with both black seed oil (0.5 ml/kg) + vitamin E (200 mg/kg) daily. Both the black seed oil and vitamin E were supplemented in feed without any vehicle. The experiment was carried out for a period of 16 weeks during which no sickness or death was recorded in any of the study groups. Doses were adjusted according to a previous study (Shahroudi et al., 2017). The present study period was considered a little bit longer than a previous study to get the desired effects of black seed oil and vitamin E on reproductive functions of mice (Parandin et al., 2012). A flow diagram of the experimental approach is presented in the Supporting Information Figure 1.

### 2.3 Collection of blood

At the end of the experiment, blood samples were collected by sacrificing the mice. In so doing, the mice were kept fasting overnight. Then the mice were placed in an airtight container containing diethyl ether presoaked cotton once at a time. After a while, the mice were checked for loss of consciousness. The mice were taken out and blood sample was collected directly from heart with a sterile syringe. About 1 to 1.5 ml blood was collected and transferred into a tube without anticoagulant for serum preparation. The experimental animals were sacrificed after collecting blood.

### 2.4 Preparation of serum

The tubes containing blood were placed in a slanting position at room temperature for 1 h. Then the clot was detached from the wall of the test tube carefully and allowed to settle down. Afterward the serum was collected and clarified by centrifuging at 3000 rpm for 15 min and stored at −20°C.

### 2.5 Biochemical and hormonal assays

The following hormonal parameters: serum testosterone, serum oestradiol and serum thyroxin (T4) were determined by using the testosterone radioimmunoassay kit (Berthold, Germany), oestradiol radioimmunoassay kit (Berthold, Germany), and T4 radioimmunoassay Kit (Berthold, Germany), respectively at the Institute of Nuclear Medicine & Allied Sciences (INMAS), Mymensingh Medical College, Mymensingh, Bangladesh using the standard protocol.

### 2.6 Collection of tissues for histology

The testis and ovary from each group of male and female mice, respectively were collected after complete removal of blood by perfusion with phosphate buffered saline and kept in 10% neutral buffered formalin. The well‐fixed tissues were processed, sectioned and stained as per standard procedure described by Banchroft et al. (1996) in collaboration with the Department of Pathology, Faculty of Veterinary Science, Bangladesh Agricultural University, Mymensingh. The stained slides were observed under ZEISS Primo star microscope (Germany).

### 2.7 Histoarchitecture of testes and ovary

For the male mice the section was observed under 20x objectives, photographed and from three different focuses of which diameter of 15 seminiferous tubules were measured and calculated using a scale. For the female mice different focuses were observed for visible changes of different stages of follicles in the ovary.

To evaluate spermatogenesis, seminiferous tubules were scored by means of the Johnsen score (Johnsen, 1970; Moghimian et al., 2017). A total of 20 cross‐sections of seminiferous tubules from each sample were studied at 40× magnification and the seminiferous tubules were scored on a scale of 1–10: 10: complete spermatogenesis and perfect tubules; 9: many spermatozoa present but disorganized spermatogenesis; 8: only a few spermatozoa present; 7: no spermatozoa but many spermatids present; 6: only a few spermatids present; 5: no spermatozoa or spermatids present but many spermatocytes present; 4: only a few spermatocytes present; 3: only spermatogonia present; 2: no germ cells present and 1: neither germ cells nor Sertoli cells present.

### 2.8 Statistical analysis

All data were placed and stored in Microsoft Excel‐ 2007 and imported to the software GraphPad Prism 5.0 for analysis using the one‐way ANOVA with Bonferroni multiple comparison test, *p ≤ 0.05, **p ≤ 0.01, ***p ≤ 0.001.

## 3 RESULTS

### 3.1 Effects of black oil seed and vitamin E on reproductive parameters of male mice

**FIGURE 1:**
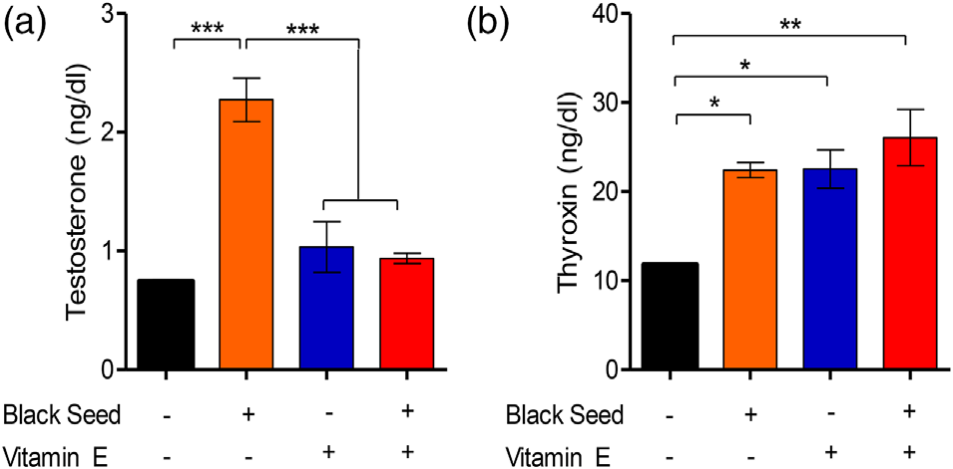
Effect of black seed oil and vitamin E and black seed oil + Vitamin E on testosterone (a) and thyroxin (b) production in male mice. Data indicate mean ± SEM of 3 replicates. Each replicate contains pooled sera of three mice. One way ANOVA with Bonferroni multiple comparison test, *p ≤ 0.05, **p ≤ 0.01, ***p ≤ 0.001

The effect of black seed oil and vitamin E on hormonal profiles and histoarchitecture of testes of Swiss Albino mice was investigated. Mice fed with normal ration (control, Group A) or mice fed with black seed oil, vitamin E or both (treatment, Group B–D) for 16 weeks were analyzed. The study revealed that serum testosterone concentration was 0.74 ± 0.01 ng/dl in group A (control) which was significantly increased in mice treated with black seed oil (group B, 2.27 ± 0.18 ng/dl). Mice fed with vitamin E (group C, 1.03 ± 0.21 ng/dl) and combination of black seed oil and vitamin E (group D, 0.94 ± 0.04 ng/dl) also showed some elevation in the testosterone concentration than the control mice but were not statistically significant (Figure 1a). Of note, mice fed with black seed oil showed a significantly higher serum testosterone than the mice fed with vitamin E and combination of both black seed oil and vitamin E. Similarly, mice fed with black seed oil, vitamin E or both showed significantly higher serum thyroxin (T4) concentration than the control mice (Figure 1b). The serum T4 concentration was 11.77 ± 0.13 ng/dl in control mice, and 22.43 ± 0.83 ng/dl, 22.54 ± 2.14 ng/dl and 26.06 ± 3.15 ng/dl in mice fed with black seed oil, vitamin E and combination of black seed oil and vitamin E, respectively. The highest T4 value was recorded in combined black seed oil and Vitamin E (group D) treated mice.

**FIGURE 2:**
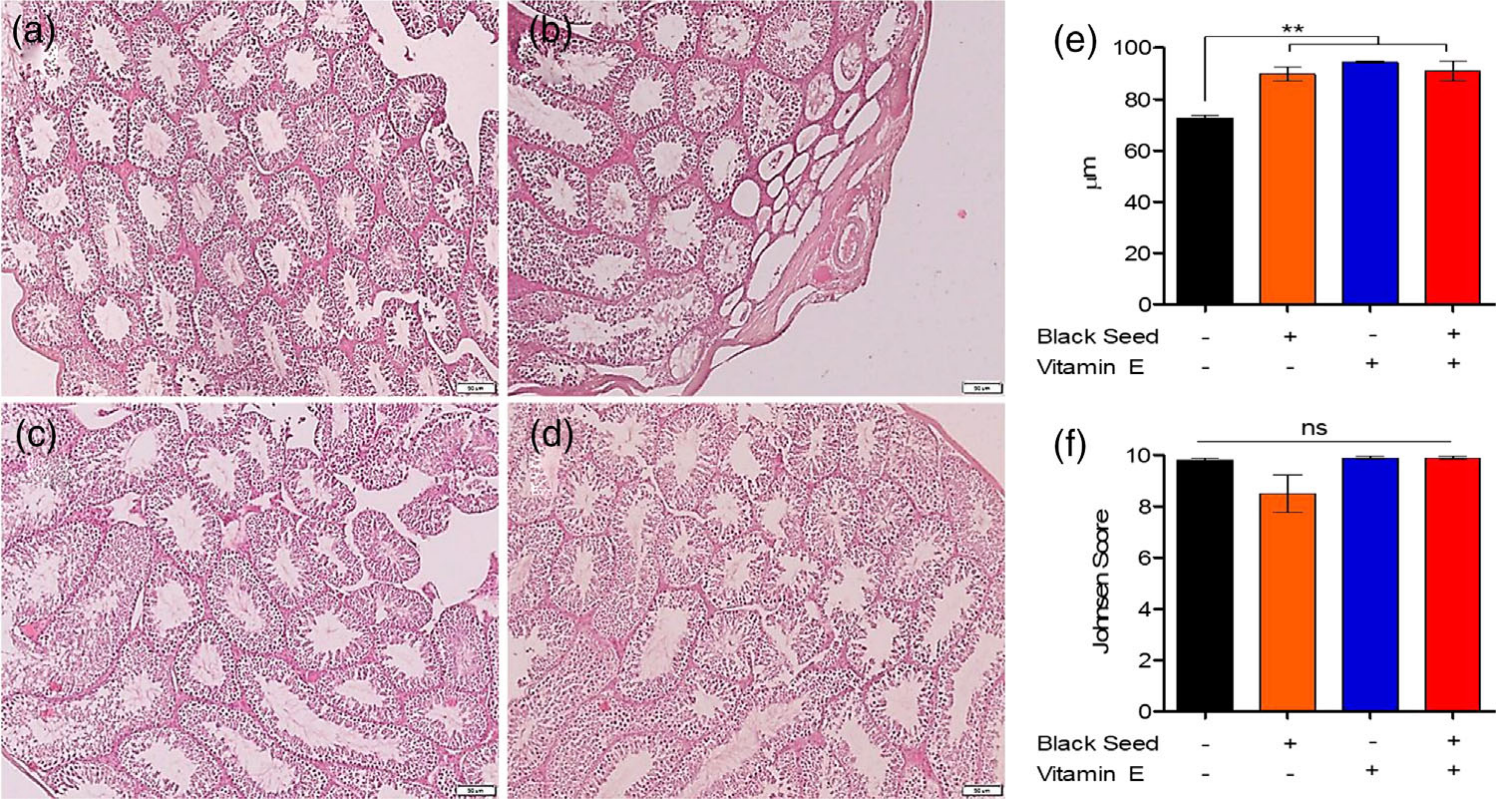
Effects of black seed oil and vitamin E on development of seminiferous tubules in mice. Photomicrograph of testes of control and treated mice showing an increase in the diameter of seminiferous tubules in mice treated with black seed oil (b), vitamin E (c) and black seed oil + vitamin E (d). Bar = 50 μm indicate magnification (a–d). The diameter of seminiferous tubules in mice showing in micrometer (e). The mean Johnsen score (MJS) of control and treated mice (f). Data indicate mean ± SEM. One way ANOVA with Bonferroni multiple comparison test, ns = not significant, *p ≤ 0.05, **p ≤ 0.01, ***p ≤ 0.001

Later on, we studied the effect of black seed oil and vitamin E supplementation on the histoarchitecture of testes of mice. To this end, we collected the testes of mice from both control and treatment groups and analyzed histology. Overall, testes from all four groups of mice showed similar histoarchitecture without obvious changes (Figure 2a–d). To get more insight into the histoarchitecture, we quantified the diameter of the seminiferous tubules from the four different groups as shown in Figure 2a–d. The diameters of seminiferous tubules of mice treated with black seed oil, vitamin E and both black seed oil and vitamin E showed significantly higher diameters than the control mice. The diameter of the seminiferous tubules of control mice was 72.57 ± 1.04 μm. On the other hand, the diameter of seminiferous tubules in mice treated with black seed oil, vitamin E and combination of black seed oil and vitamin E were 89.77 ± 2.6 μm, 94.37 ± 0.40 μm and 90.89 ± 3.82 μm, respectively.

Finally, the histoarchitecture and functionality of the seminiferous tubules were evaluated using the mean Johnsen score (MJS) (Banchroft et al., 1996). A total of 20 cross‐sections of seminiferous tubules from each group as shown in Figure 2a–d were studied at 40× magnification and scored. The control mice had MJS of 9.8 ± 0.09 while mice receiving black seed oil, vitamin E and combination of black seed oil and vitamin E had MJS of 8.5 ± 0.73, 9.9 ± 0.06 and 9.9 ± 0.06, respectively. However, there was no significant differences in the MJS of the four different treatment groups (Figure 2f).

### 3.2 Effects of black oil seed and vitamin E on reproductive parameters of female mice

**FIGURE 3:**
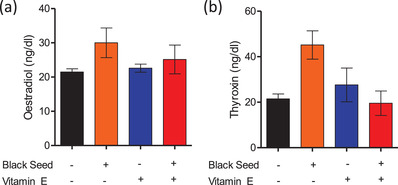
Effect of black seed oil, vitamin E and black seed oil + Vitamin E on oestradiol (a) and thyroxin (b) production in female mice. Data indicate mean ± SEM of three replicates. Each replicate contains pooled sera of three mice. One way ANOVA with Bonferroni multiple comparison test, *p ≤ 0.05, **p ≤ 0.01, ***p ≤ 0.001

In female mice, the supplementation of black seed oil and combination of black seed oil and vitamin E showed an elevated concentration of serum oestradiol level than the control mice, however, was not statistically significant (Figure 3a). The serum oestradiol concentration of control mice was 21.47 ± 0.94 ng/dl. Mice received black seed oil, vitamin E and combination of black seed oil and vitamin E showed serum oestradiol concentration of 30.03 ± 4.34 ng/dl, 22.6 ± 1.17 ng/dl and 25.16 ± 4.19 ng/dl, respectively. Similarly, mice received black seed oil (45.16 ± 6.22) also showed an elevated concentration of serum thyroxin (T4) level than the control mice (21.49 ± 2.12 ng/dl), however, was statistically insignificant (Figure 3b). Mice received the vitamin E (27.6 ± 7.45 ng/dl) and combination of black seed oil and vitamin E (19.55 ± 5.36) showed T4 level comparable to the control mice.

**FIGURE 4:**
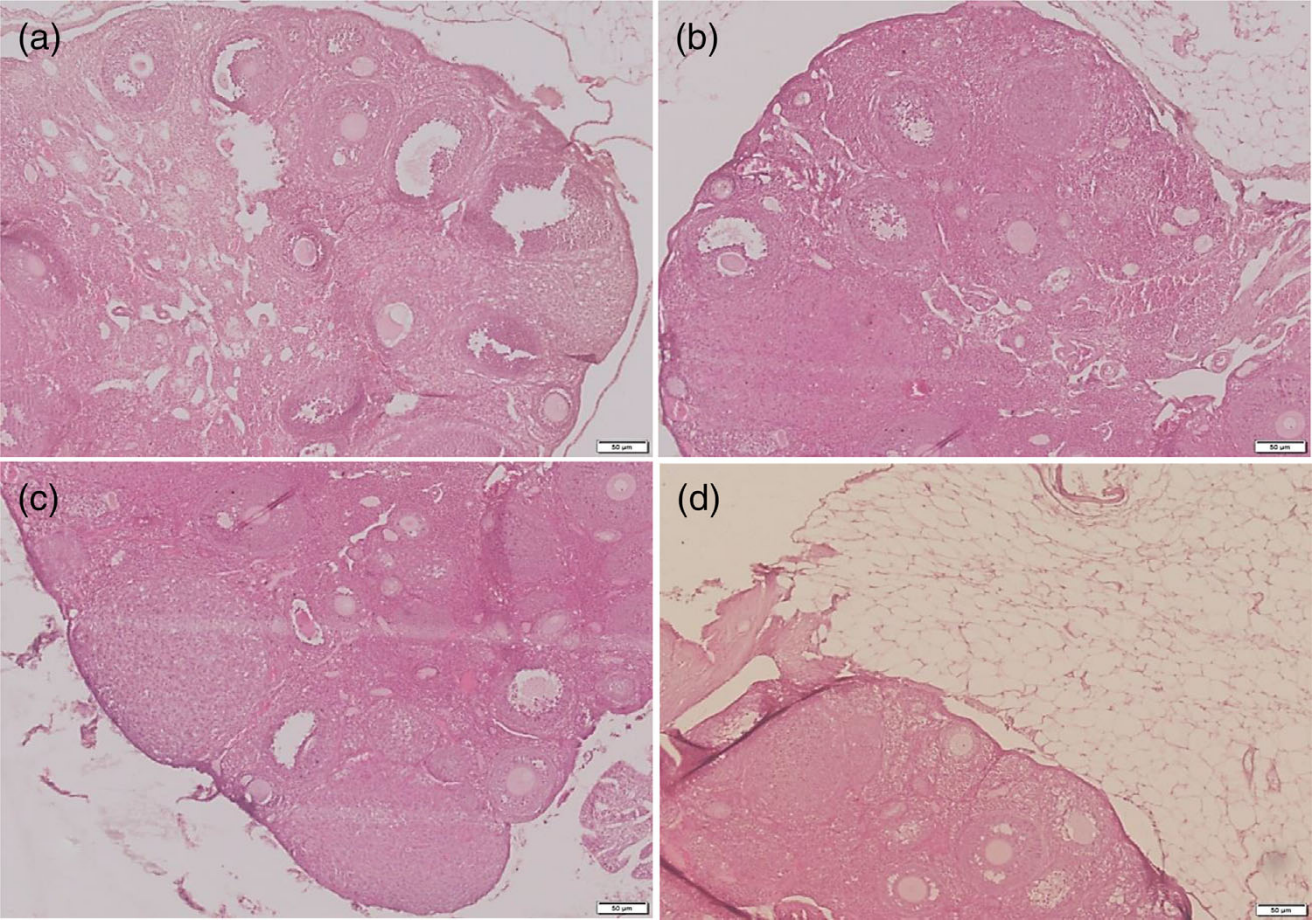
Effects of black seed oil and vitamin E on ovary in female mice. Photomicrograph of ovary of control (a) and treated mice (b–d) showing an increase in the number of follicles in mice treated with black seed oil (b), vitamin E (c) and black seed oil + vitamin E (d). Bar = 50 μm indicate magnification (a–d)

The histological section of ovary of mice supplemented with black seed oil and vitamin E was analyzed. The result showed an increase of the follicles of different stages in mice supplemented with black seed oil, vitamin E and combined black seed oil and vitamin E as compared to the control (Figure 4). Taken together, the black seed oil and vitamin E supplementation showed a positive response in increasing reproductive hormones as well as improving folliculogenesis in ovary of female mice.

## 4 DISCUSSIONS

Oxidative stress damages the reproductive system and sperm, and therefore results in reducing sperm motility, lipid peroxidation and oocyte‐sperm fusion in addition to increasing the DNA damage (Ko et al., 2014; Shokoohi et al., 2018). ROS has harmful effects on motility, morphology and concentration of sperm and it causes sperm DNA damage and apoptosis (Ko et al., 2014; Shokoohi et al., 2018).

This research demonstrates that oral administration of *N. sativa* doses 0.5 ml/kg and vitamin E doses 200 mg/kg body weight in Swiss albino mice for 16 weeks caused a significant increase in some fertility parameters. The testes, epididymis and other reproductive organs are structurally and physiologically dependent upon the testosterone and other androgens. Testosterone stimulates growth and secretary activity of the reproductive organs (Singh et al., 1995), therefore, a significant increase of these hormones in our study could increase the number and function of somatic and germinal cells of the testis. In the present study mice supplemented with black seed oil had a significant increase in the testosterone levels. Our results are in agreement with two previous studies of Mukhalad et al. (2009) and Gokçe et al. (2010). The phytochemical analytical study of Nickavar and colleagues indicated the rich presence of unsaturated fatty acids (linoleic acid 55.6%, oleic acid 23.4%, palmitic acid 12.5%, stearic acid 3.4% and else.) in *N. sativa* seeds (Nickavar et al., 2003). In study of Fellner and colleagues, the dietary supplementation of rats with oils rich in polyunsaturated fatty acids, such as linoleic acid has positively influenced reproductive functions (Fellner et al., 1995). The study of Gromadzka‐Ostrowska and colleagues also showed that the unsaturated fatty acids stimulate the activity of 17 β‐hydroxysteroid dehydrogenase, the most important key enzyme in the testosterone biosynthesis pathway (Gromadzka Ostrowska et al., 2002). In this connection Ayan et al. (2016) has recently reported that thymoquinone which is the main constituents of black seed oil has an antioxidant activity and decreases superoxide dismutase, glutathione peroxidase and malondialdehyde levels in testicular tissues. This plays a protective role against oxidative injury of testis in rats (Shittu et al., 2013). The intake of vitamin E has shown to significantly increase the amount of testosterone concentration too. The study also showed that vitamin E can repair the quality of the sperm cells, as well as the diameter of seminiferous tubules (Elayapillai et al., 2017). Similarly, Momeni et al. (2012) showed that vitamin E significantly improved sperm quality and ameliorated the testicular morphologic parameters in sodium arsenite‐treated rats. Moreover, it has been reported that vitamin E improves plasma level of gonadal hormones and enhances fertilizing capacity in noise‐stressed rats (Rajabzadeh et al., 2015). According to a study by Al‐Kushi et al. (2016) vitamin E can be effective in treating erectile dysfunction, hormonal imbalance and oligospermia, all of which can increase fertility. Vitamin E has been shown to be effective as an antioxidant in fighting against external and toxic factors in testicular tissue (Erdamar et al., 2008). The present findings were also supported by Bashandy who stated that levels of testosterone and follicle stimulating hormone (FSH) in testicular tissues are increased due to phenolic and alkaloid compounds (Bashandy, 2007). Overall, the effects of the black seed oil and vitamin E on the reproductive functions could vary depending on the dosages and duration of the treatment. However, most of the human trials of black seed oil also used doses similar to this study (Hadi et al., 2021; Koshak et al., 2021).

In the present study we did not find any significant changes of oestrogen concentration in female mice treated with black seed oil and vitamin E although the follicular growth was increased. But a previous study revealed that black seed oil has a positive effect on oestrogen concentration due to its content thymoquinone and unsaturated fatty acids (Liu et al., 2004). Parhizkar et al. studied that linoleic acid, an active ingredient of *N. sativa* has estrogenic effects, which increases gradually the blood levels oestrogen, and increases vaginal epithelial cells (Parhizkar et al., 2016).

Kamarzaman and colleagues demonstrated the prophylactic effect of *N. sativa* on the number of ovarian follicles and diameters against cyclophosphamide in adult mice (Kamarzaman et al., 2014). Our results also supported by Arif et al. (2016) who recently employed in vitro and in vivo models to investigate the effect of thymoquinone on ovarian morphology and function. Our results showed that when black seed oil and vitamin E supplemented alone the fertility parameters improved, however after coupled administration they worked synergistically.

On the other hand, thyroid disease, constitutes the most common endocrine abnormality in recent years, diagnosed either in subclinical or clinical form and is associated with various metabolic abnormalities, due to the effects of thyroid hormones on nearly all major metabolic pathways (Grzanna et al., 2005), increasing the basal metabolic rate, affecting protein synthesis, regulating the metabolism of protein, lipids and carbohydrates and involved in the regulation of oxidative metabolism (Chu, 2005). Thyroid hormones can cause many changes in the number and activity of mitochondrial respiratory chain components. This may results in the increase generation of ROS (Saleh, 2015). Some studies showed an increase production of ROS in hypothyroidism (Werner et al., 2005). The increase concentration of thyroxin in black seed oil, vitamin E and black seed oil + vitamin E treated mice was also recorded in the present study for male mice. But for the female mice the value showed no significant changes. A previous study reported an increased T4 levels in rabbits receiving oral administration of black seed oil (Sharif et al., 2012).

A recent study identified the thyroid hormone receptors (TRs) directly on the testes and showed that thyroid hormone affects the growth and development of the male testes (Panahi et al., 2011). Similarly, thyroid hormones are vital for the proper functioning of the female reproductive system, since they modulate the metabolism and development of ovarian, uterine and placental tissues. Therefore, hypo‐ and hyperthyroidism may result in subfertility or infertility in women. Therefore, the black seed oil and vitamin E can be useful in treating disorders associated with hypo‐ and hyperthyroidism.

## 5 CONCLUSIONS

From the observation in this study, we conclude that the oral administration of black seed oil and vitamin E showed a positive effect on reproductive performance of mice and increases fertility power when administrated separately. But when applied together the reproductive performance did not reveal adequate improvement. Our study suggests that black seed oil and vitamin E can be useful to treat various infertility and reproductive disorders. The scanning electron microscopy should be carried out to get more accurate pictures of testis and ovary. However, the detailed mechanism of action of black seed oil and vitamin E demands further studies. The study was limited to a single dose of these supplements and was applied to healthy mice. Therefore, the enhancing effects of these supplements on reproductive parameters should be testing using different dosages and with specific disease models.

## CONFLICT OF INTEREST

The authors declare no conflict of interest.

## ETHICAL APPROVAL

Animal care, preparation and experimental protocols were approved by the Animal welfare and Experimentation Ethics Committee of Bangladesh Agricultural University, Mymensingh. All animals were managed in a manner consistent with the Policy for Animal research (Ref. No. AWEEC/BAU/2020 (31), Date; 20.10.2020).

## AUTHOR CONTRIBUTIONS

**Afrina Mustari**: Conceptualization; Data curation; Formal analysis; Funding acquisition; Investigation; Writing original draft; Writing review & editing. **Mohammed Nooruzzaman**: Data curation; Formal analysis; Investigation; Software; Writing original draft; Writing review & editing. **Mohammad Alam Miah**: Data curation; Formal analysis; Writing original draft. **Khaled Mahmud Sujan**: Data curation; Formal analysis. **Emdadul Haque Chowdhury**: Data curation; Formal analysis; Writing review & editing

### PEER REVIEW

The peer review history for this article is available at https://publons.com/publon/10.1002/vms3.708

## Supporting information

FigureS1

## Data Availability

The data that support the findings of this study are available from the corresponding author upon reasonable request.
