# Supplementary figures and images for: Promoting action of vitamin E and black seed oil on reproductive hormones and organ histoarchitecture of Swiss albino mice
Source: Vet Med Sci. 2022 Jan 17;8(2):710–8. doi: 10.1002/vms3.708 (PMC8959291; doi:10.1002/vms3.708)

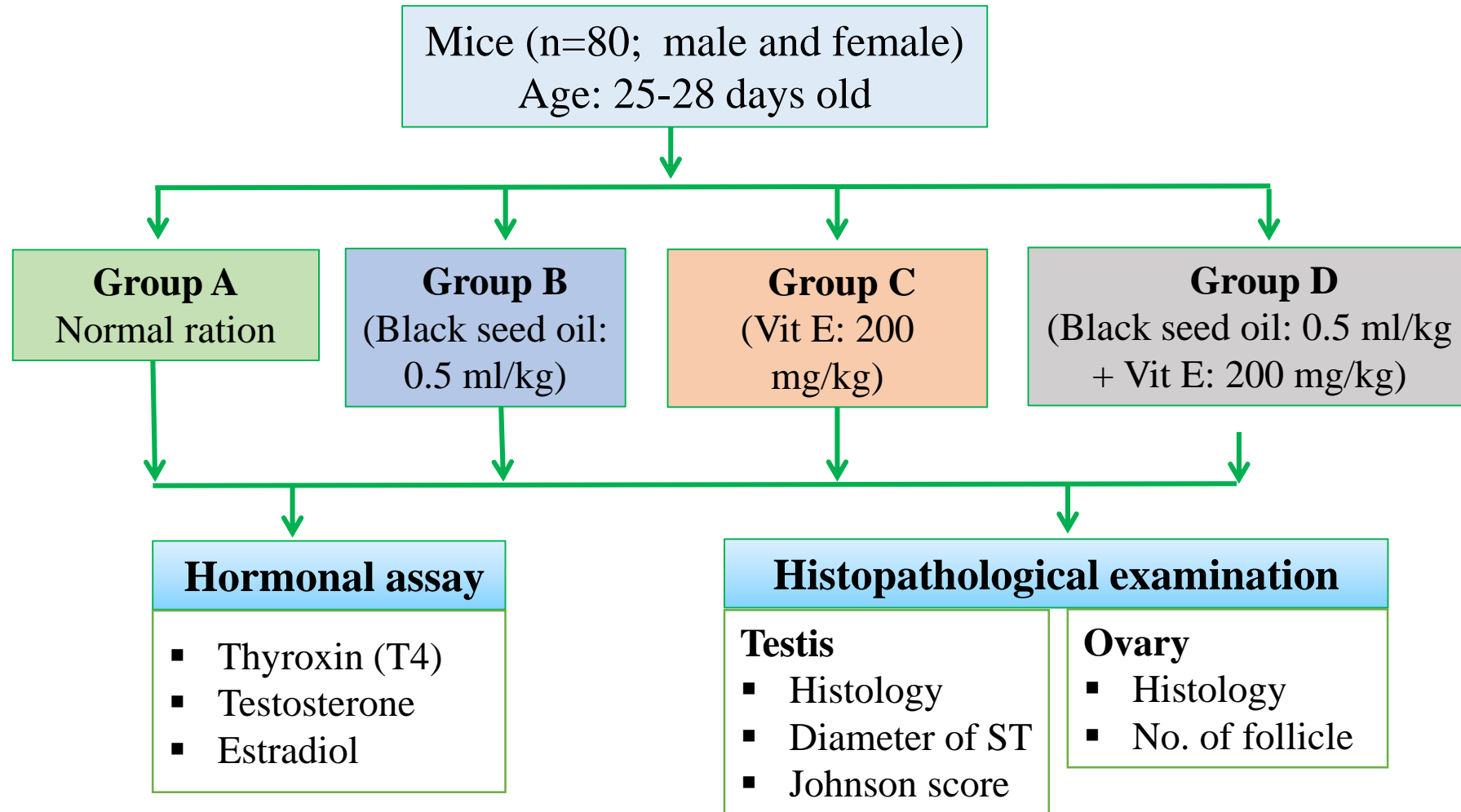

Supplement: Supplementary file 1 — FigureS1 [file VMS3-8-710-s001.pdf]
